# Complete F mitochondrial genomes of two freshwater mussels from the Lake Biwa system in Japan: *Beringiana fukuharai* and *Sinanodonta tumens*

**DOI:** 10.1080/23802359.2021.1955762

**Published:** 2021-07-26

**Authors:** Kohji Mabuchi, Kazuya Nishida, Nobuyoshi Nakajima

**Affiliations:** a Lake Biwa Branch Office, National Institute for Environmental Studies, Otsu, Japan; b Environmental Genomics Office, National Institute for Environmental Studies, Tsukuba, Japan

**Keywords:** F mitogenome, *Beringiana fukuharai*, *Sinanodonta tumens*, Lake Biwa, Illumina sequencing

## Abstract

We determined the complete mitochondrial sequences of female-transmitted (F) mitogenomes of six unionid specimens from the Lake Biwa system, Japan. Their gene contents and orders agreed with those of the typical F mitogenome of freshwater mussels. Molecular phylogenetic analysis using fifteen previously identified partial COI and 12 (six previously identified and six newly determined) whole mitogenome sequences revealed that five of the six mitogenomes (LC592401, LC592402, LC592403, LC592408, and LC592410) were those of *Beringiana fukuharai*, while the remaining one (LC592406) was *Sinanodonta tumens*.

Freshwater mussels (Bivalvia: Unionidae) are burrowing, filter-feeding bivalves. They occur in lakes and rivers worldwide, but are now declining in many countries (Lopes-Lima et al. 2014, 2018). Conservation actions have been hindered by difficulties with species recognition and identification (Ferreira-Rodríguez et al. 2019) due to the high plasticity of shell shape within species and its convergence between species (Klishko et al. 2016). Recent molecular analyses are resolving the difficulties, and Lopes-Lima et al. (2020) recently proposed a new classification for Unionidae from Far East Asia, based on COI + 28S phylogenies. This separated the genus *Beringiana* from the genus *Sinanodonta* and established two new *Beringiana* species: *Beringiana fukuharai* Sano, Hattori and Kondo in Lopes-Lima et al. (2020) and *B. gosannensis* Sano, Hattori and Kondo in Lopes-Lima et al. (2020).

Previously, we developed a DNA mini-barcoding system for unionids from the Lake Biwa system in Japan (Mabuchi and Nishida 2020). A single primer set was designed to amplify an approximately 140-bp barcode fragment within the mitochondrial 16S rRNA gene region, and reference DNA sequences for species identification were obtained using these primers. To adapt this barcoding system to the new classification, we determined the mitogenome sequences of six selected specimens called ‘*Sinanodonta*’ in the barcoding system (specimen IDs B57, B103, B177, B194, n972, and n997 in Mabuchi and Nishida 2020), and re-identified each specimen through a phylogenetic analysis of the 6 mitogenomes and 15 previously identified partial COI sequences of the new *Sinanodonta* and *Beringiana* used in Lopes-Lima et al. (2020). To know the phylogenetic relationship among the six mitogenomes more reliably, five additional *Sinanodonta* mitogenomes, which were ranked among the six mitogenomes in the BLAST searches, were included in the phylogenetic analysis: one mitogenome of *Sinanodonta lucida* (Heude, 1878) [accession no. KF667529 used in Song et al. (2016), registered as *Anodonta lucida* in DNA databases] and four mitogenomes of *Sinanodponta woodiana* (Lea, 1834) [HQ283344 and HQ283345, the latter used in Soroka (2010), registered as *Anodonta woodiana* in DNA databases, KM272949 used in Zhang et al. (2016), and MN594536 used in Riccardi et al. (2020)]. The *Cristaria plicata* (Leach, 1814) mitogenome [GU944476 used in Lee et al. (2012)] was used as an outgroup, because the genus *Cristaria* occupied a basal position within the tribe Cristariini (including the above species) in the molecular phylogeny of Lopes-Lima et al. (2020).

The six specimens sequenced here were deposited in the Lake Biwa Museum, Shiga Prefecture, Japan (https://www.biwahaku.jp/, Masanari Matsuda, matsuda-masanari@biwahaku.jp) under registration numbers LBM-1300014502, 1300014522, 1300014527, 1300014538, 1300005351, and 1300014554 (for correspondence with specimen ID, see Figure 1). The tissue (& DNA) samples were stored in the Lake Biwa Branch Office, National Institute for Environmental Studies, Shiga Prefecture, Japan (http://www.nies.go.jp/biwakobranch/, Kohji Mabuchi, mabuchi.koji@nies.go.jp) under registration numbers used here as the specimen IDs. Genomic DNA was isolated from foot muscle tissue and sequenced using Illumina MiSeq and HiSeq X Ten sequencers (Illumina). The resultant reads were assembled using CLC Genomic Workbench (ver. 11.01; QIAGEN), and low coverage regions were confirmed by Sanger sequencing with AB 3130xl genetic analyzer (Applied Biosystems). The obtained mitogenome sequences were annotated by alignment with the two *Sinanodonta* mitogenomes (KF667529 and KM272949). Using the 27 sequences mentioned above, we constructed a phylogenetic tree using a supermatrix approach (de Queiroz and Gatesy 2007), as described in Figure 1.

**Figure 1.**
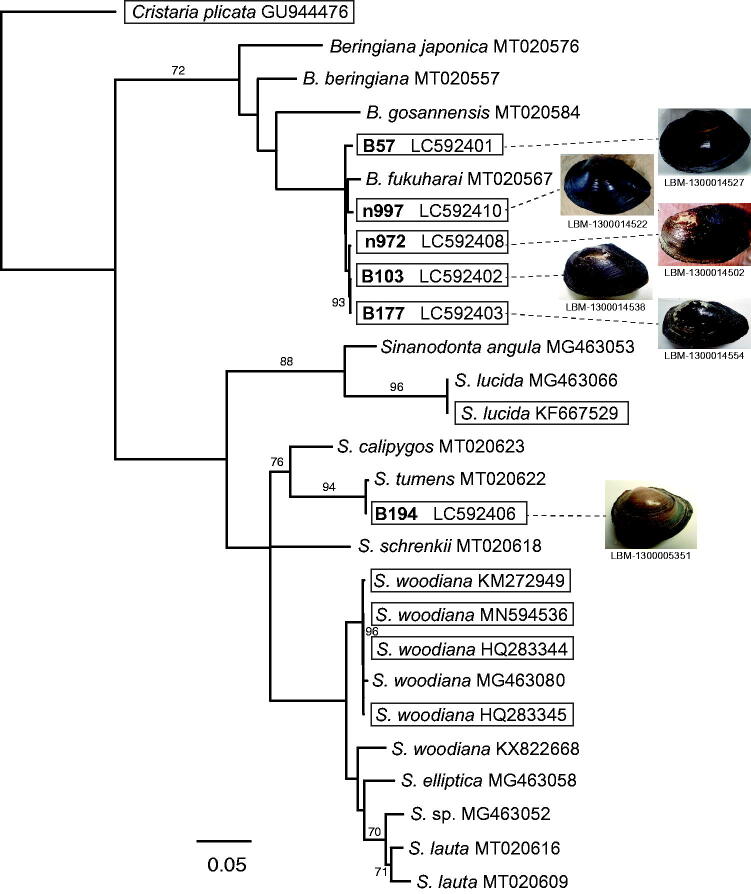
Supermatrix tree of 12 mitogenomes (15,708–16,385 bp) and 15 partial COI sequences (597–657 bp) of female-transmitted (F) mitogenomes of *Sinanodonta* and *Beringiana* species (*Cristaria plicata* used as an outgroup). Bootstrap support (≥70%) is indicated at the nodes. For the previously identified sequences (15 partial sequences and six mitogenomes), accession numbers are given after the species names. For the six mitogenomes sequenced here, the accession numbers are indicated after the specimen IDs used in Mabuchi and Nishida (2020) (the IDs are in bold). The six newly sequenced and six previously published mitogenomes are boxed. The tree backbone was first generated for the 12 mitogenomes by the neighbor-joining (NJ) method using the online version of MAFFT (https://mafft.cbrc.jp/alignment/server/). The obtained NJ tree was then used as a backbone constraint for the supermatrix tree, which was constructed based on the dataset including the 12 mitogenomes and 15 partial sequences, which were first aligned using MAFFT and corrected by eye using Mesquite (version 3.31; http://www.mesquiteproject.org). After deleting the intergenic region, maximum likelihood analysis was performed for the resultant 14,785-bp dataset using RAxML BlackBox (https://embnet.vital-it.ch/raxml-bb/).

All six mitogenomes determined here (DDBJ accession nos. LC592401, LC592402, LC592403, LC592406, LC592408, and LC592410; for correspondence with specimens, see Figure 1) contained 13 protein-coding, 2 rRNA, and 22 tRNA genes, and their orders in the mitogenome were those typical of female (F)-transmitted freshwater mussel mitogenomes (Breton et al. 2009). For all the six mitogenomes, one open reading frame (ORF) spanning 255 or 267 nucleotides, was recognized in the region between tRNA-Glu and ND2 genes, which seemed to correspond to the female-specific ORF described in Breton et al. (2009).

The resulting supermatrix tree (Figure 1) contained two major clades: *Beringiana* and *Sinanodonta*. The phylogenetic positions of the six mitogenomes indicated that five (LC592401, LC592402, LC592403, LC592408, and LC592410) were those of *Beringiana fukuharai*, while the remaining one (LC592406) was *Sinanodonta tumens* (Haas, 1910). The five specimens identified here as *B. fukuharai* had been identified as *Sinanodonta japonica* (von Martens, 1874) (B103, B177, n972) or *Sinanodonta calipygos* (Kobelt, 1879) (B57, n997) based on shell morphology in Mabuchi and Nishida (2020) (for the shell shape of each specimen, see pictures in Figure 1). The identification of the remaining specimen [identified as *Sinanodonta ogurae* (Kuroda and Habe, 1987) in Mabuchi and Nishida 2020] was confirmed by our analysis: the scientific name ‘*Sinanodonta ogurae*’ was synonymized under *S. tumens* in Lopes-Lima et al. (2020).

## Geolocation information

35.449783N, 136.196045E (B103); 35.249828N, 136.210316E (B177); 35.449774N, 136.196045E (n972); 35.027062N, 135.869484E (n997); 35.225730N, 136.159840E (B57); 35.015376N, 135.946618E (B194).

## Data Availability

The genome sequence data supporting this study are openly available in NCBI and DDBJ at nucleotide database, https://www.ncbi.nlm.nih.gov/nuccore/LC592401, LC592402, LC592403, LC592408, LC592410, LC592406, Associated BioProjects, https://www.ncbi.nlm.nih.gov/bioproject/PRJDB10947, PRJDB11033, PRJDB11034, PRJDB11035, PRJDB11036, PRJDB11037, BioSample accession numbers at https://www.ncbi.nlm.nih.gov/biosample/SAMD00265731, SAMD00271493, SAMD00271494, SAMD00271495, SAMD00271496, SAMD00271503, and Sequence Read Archives at https://ddbj.nig.ac.jp/DRASearch/submission?acc=DRA011399, DRA011400, DRA011401, DRA011402, DRA011403, DRA011404, DRA011405, DRA011406, DRA011407, DRA011408, DRA011409, DRA011410, DRA012100, DRA012101, DRA012102, DRA012103, DRA012104, DRA012105.
